# Promoting sexual and reproductive health among adolescents in southern and eastern Africa (PREPARE): project design and conceptual framework

**DOI:** 10.1186/1471-2458-14-54

**Published:** 2014-01-18

**Authors:** Leif Edvard Aarø, Catherine Mathews, Sylvia Kaaya, Anne Ruhweza Katahoire, Hans Onya, Charles Abraham, Knut-Inge Klepp, Annegreet Wubs, Sander Matthijs Eggers, Hein de Vries

**Affiliations:** 1 Division of Mental Health, Norwegian Institute of Public Health, Oslo, Norway; 2 Department of Health Promotion and Development, Faculty of Psychology, University of Bergen, Bergen, Norway; 3 Health Systems Research Unit, Medical Research Council, Cape Town, South Africa; 4 Adolescent Health Research Unit, Department of Psychiatry and Mental Health, University of Cape Town, Cape Town, South Africa; 5 Department of Psychiatry and Mental Health, Muhimbili University of Health and Allied Sciences, Dar es Salaam, Tanzania; 6 Child Health and Development Centre, College of Health of Science, Makerere University, Kampala, Uganda; 7 Department of Public Health Practice and Health Promotion, School of Health Sciences, University of Limpopo, Turfloop Campus, Sovenga, South Africa; 8 University of Exeter Medical School, Exeter, UK; 9 Institute of Basic Medical Sciences, Faculty of Medicine, University of Oslo, Oslo, Norway; 10 Department of Health Education and Health Promotion, Maastricht University, Maastricht, The Netherlands

## Abstract

**Background:**

Young people in sub-Saharan Africa are affected by the HIV pandemic to a greater extent than young people elsewhere and effective HIV-preventive intervention programmes are urgently needed. The present article presents the rationale behind an EU-funded research project (PREPARE) examining effects of community-based (school delivered) interventions conducted in four sites in sub-Saharan Africa. One intervention focuses on changing beliefs and cognitions related to sexual practices (Mankweng, Limpopo, South Africa). Another promotes improved parent-offspring communication on sexuality (Kampala, Uganda). Two further interventions are more comprehensive aiming to promote healthy sexual practices. One of these (Western Cape, South Africa) also aims to reduce intimate partner violence while the other (Dar es Salaam, Tanzania) utilises school-based peer education.

**Methods/design:**

A modified Intervention Mapping approach is used to develop all programmes. Cluster randomised controlled trials of programmes delivered to school students aged 12–14 will be conducted in each study site. Schools will be randomly allocated (after matching or stratification) to intervention and delayed intervention arms. Baseline surveys at each site are followed by interventions and then by one (Kampala and Limpopo) or two (Western Cape and Dar es Salaam) post-intervention data collections. Questionnaires include questions common for all sites and are partly based on a set of social cognition models previously applied to the study of HIV-preventive behaviours. Data from all sites will be merged in order to compare prevalence and associations across sites on core variables. Power is set to .80 or higher and significance level to .05 or lower in order to detect intervention effects. Intraclass correlations will be estimated from previous surveys carried out at each site.

**Discussion:**

We expect PREPARE interventions to have an impact on hypothesized determinants of risky sexual behaviour and in Western Cape and Dar es Salaam to change sexual practices. Results from PREPARE will (i) identify modifiable cognitions and social processes related to risky sexual behaviour and (ii) identify promising intervention approaches among young adolescents in sub-Saharan cultures and contexts.

**Trial registrations:**

Controlled Trials ISRCTN56270821 (Cape Town); Controlled Trials ISRCTN10386599 (Limpopo); Clinical Trials NCT01772628 (Kampala); Australian New Zealand Clinical Trials Registry ACTRN12613000900718 (Dar es Salaam).

## Background

With an estimated 68 per cent of all HIV-infected people, sub-Saharan Africa remains the region of the world which is most severely affected by this pandemic [1,2]. Among the approximately 5 million young people (age 15–24) who lived with AIDS worldwide in 2008, 80% lived in sub-Saharan Africa [1]. The incidence of HIV infections is presently decreasing in a number of countries in sub-Saharan Africa, and this decrease takes place also among young people [1]. Nonetheless, the challenge is daunting. Preventive programmes must target adults as well as adolescents. Preventive programmes targeting adolescents have been shown to be more effective if they take an integral approach that includes several stakeholders at the level of the broader environment (e.g. teachers, health personnel, parents), and ideally also includes action to influence macro level factors such as policies that facilitate the implementation of programs and promote health [3,4]. With respect to HIV prevention, studies have shown that interventions targeting adolescents are more effective if they target sexually inexperienced youth [5,6]. Interventions in general, and thus also HIV prevention interventions, should be research based and evaluated in studies with rigorous research designs and data collection instruments of high quality [7].

Behavioural interventions targeting adolescents may contribute to reducing the incidence of HIV infections in three ways: (i) by postponing sexual debut among those who are sexually inexperienced, (ii) promoting consistent use of condoms, and (iii) reducing the number of concurrent sexual partners [1,8]. The purpose of this article is to present theoretical frameworks, intervention development and methods used in a project aiming to evaluating four interventions targeting social cognitive mediators and behaviours related to sexual- and reproductive health among adolescents at four sites in sub-Saharan Africa.

### The PREPARE project

A consortium consisting of researchers from eight universities, four African and four European, succeeded in obtaining support from the European Commission for a 54 month project which includes evaluation of four interventions targeting adolescents in their early teens at four sites in sub-Saharan Africa: Western Cape and Mankweng in Limpopo (South Africa), Dar es Salaam (Tanzania), and Kampala (Uganda). The overall purpose of this research project is to develop and evaluate interventions which are effective in reducing the spread of sexually transmitted diseases (including HIV) and unwanted pregnancies by changing sexual- and reproductive behaviour and determinants of such behaviour. Another purpose is to examine the utility of social cognition models in predicting sexual behaviour in cultural contexts where there have been few studies examining their relevance [9].

Risky sexual practices have been found to be influenced by a range of personal, social and contextual factors [10], thus indicating the need to address a range of factors in HIV-preventive interventions. PREPARE aims to develop and test comprehensive school-based community prevention approaches in two sites (Western Cape and Dar es Salaam) and more focussed school-based interventions in two other sites (Limpopo and Kampala). The two comprehensive interventions are designed to influence sexual behaviour as well as hypothesized mediators representing underlying change processes (i.e. knowledge, attitudes, norms, self-efficacy and intentions, interpersonal relationships and violence in intimate relationships). The two more focussed interventions were designed to test interventions designed to change specified determinants based on previously applied social cognitive models. All interventions target young people at an age when most of them are yet to establish habitual sexual behaviour patterns.

### Study objectives

The PREPARE project grew out of a consortium which has previously conducted a multi-site intervention project in sub-Saharan Africa, the SATZ project [11]. The SATZ interventions were similar across sites, although local adjustments were made in order to adjust to local circumstances and meet local needs. An important lesson learned from the SATZ study was that intervention effects varied widely across sites, and that contextual factors rather than content and delivery of interventions could explain these differences [12].

The PREPARE interventions will be developed separately. Each site begins with particular intervention objectives and develops their own intervention programme in order to meet local needs and in order to take local contexts and circumstances into account. This is expected to increase chances of successful outcomes, and it is also expected to increase insights into contextual and environmental moderation of intervention effectiveness.

The primary objective of the *Western Cape* PREPARE study is to evaluate the effects of the interventions on sexual risk behavior and interpersonal violence, and assess whether reductions in sexual risk behavior are mediated by norms and attitudes about, and perpetration of interpersonal violence. A secondary objective is to evaluate the effects of the interventions of the incidence of conceptions among female participants during the three years following the baseline survey, using records of deliveries and terminations of pregnancies collected routinely by the Western Cape public health services.

The primary objective of the *Dar es Salaam* PREPARE study is to evaluate the effects of the interventions on early sexual transition and on the frequency and quality of peer-to-peer communication on puberty and sexual risks reduction, and to assess whether effects of the intervention programme on sex transition are mediated by such communication. Secondary objectives are to evaluate the effects of the interventions on adolescent’s knowledge and attitudes about puberty, HIV/STI transmission, condom use and on the incidence and consistency of condom use among sexually active adolescents.

The primary objective of the Limpopo study is to investigate the effect of the Limpopo PREPARE school-based intervention on beliefs, attitudes, norms and culture-specific beliefs found to be associated with sexual practices increasing HIV risk among adolescents. A secondary objective is to compare the utility of modifiable cognitions specified by widely applied social cognitive models and culture-specific beliefs in predicting sexual behaviour in a cultural context in which such models have been infrequently applied.

The primary aim of the *Kampala* PREPARE study is to evaluate an intervention focussed on promoting communication between adolescents and their parents/caregivers. This includes examining effects on the frequency and quality of parent-adolescent communication on issues related to sexuality. A secondary objective is to study changes in parenting skills and parents’ attitudes towards sexuality communication with their adolescent children.

The two focussed interventions (Limpopo and Kampala), targeting modifiable determinants, are designed to be integrated into pre-existing health promotion curricula. Also in the two sites where more comprehensive community interventions are implemented, site-specific components annexed to the main interventions are included, a violence prevention component in Cape Town and a peer education component in Dar es Salaam. Both comprehensive interventions will use elements of a whole school development approach [13] and in addition involve partners beyond the school organization such as parents, youth health services, police departments and other community stakeholders.

### Theoretical framework

In order to be efficacious and effective, behavioural interventions should be based on an understanding of mechanisms and processes as described by empirically-tested theoretical frameworks. The PREPARE study borrows mainly from four main descriptions of psychological and behavioural change mechanisms: (i) Social cognition models such as Social Cognitive Theory [14], the Reasoned Action Framework [15], the Information-Motivation-Behavioral Skills (IMB) model [16] and the I-Change model [17]; (ii) ecological and contextual models of health behaviours [18,19]; (iii) research on attitude and behaviour change [20,21] and (iv) frameworks for intervention development [19].

An important distinction in prevention research is drawn between action theory and conceptual theory [22]. Action theory refers to how an intervention changes possible mediators such as knowledge, attitudes, subjective norms, self-efficacy or intentions. Conceptual theory specifies how the mediating variables affect behaviour. The Reasoned Action Framework [15] is a typical example of a conceptual theory. Theoretical perspectives explaining underlying processes related to the “induced compliance” technique for attitude change are examples of action theory [21]. MacKinnon [22] maintains that most research has focussed on conceptual theory, while less attention has been devoted to action theory. In the PREPARE project both theoretical domains are highlighted. The importance of applying basic intervention principles like involving students actively [21] and mobilizing support from parents and the larger community [23] were laid down as important premises for intervention development. These intervention principles are derived from relevant action theories.

### The PREPARE interventions

The development of comprehensive interventions (Dar es Salaam and Western Cape) is based on an earlier version of the Intervention Mapping approach, also referred to as programme matrixing [4,24]. Table 1 shows elements from the intervention planning matrix that was designed with the purpose of identifying important personal and social determinants related to one of the target behaviours, delay of sexual debut. Performance objectives describe a number of more specific skills and behaviours which will contribute to delayed sexual debut. Perceived benefits and perceived barriers, perceived social norms and self-efficacy are important determinants of behaviour and therefore also important change objectives. Action plans denote an approach to improved control over own behaviour and adds to the list of change objectives. The specific content is formulated based on earlier research and focus interviews in each site. The next step (not covered by Table 1) is to identify theoretically based strategies and practical methods for change among the target groups [25]. Subsequently, appropriate channels and resources are selected for each of these strategies. These programme development matrices are developed for each level of the intervention, meaning one for the student, one for the teachers, and one for each actor in the social environment (e.g. parents, police officers, peer educators, health workers).

**Table 1:** Example of an intervention planning matrix for learners

**Behavior to promote**	**Performance objectives**	**Pros (perceived benefits)**	**Cons (perceived barriers)**	**Perceived social norms**	**Self-efficacy**	**Action plans**
Delay of sexual debut	PO1: Learners will say no to sexual intercourse when they do not want it	1: It will increase the likelihood of realizing my life goals	1: I want to have sex	1: Religious influences – largely discouraging early sexual debut	Difficult to remain abstinent when … - I am deeply in love - I am sexually excited - I am in a committed relationship - I am using drugs or alcohol - My partner is a lot older than me	1. I will remind myself of my life goals when I get sexually excited
	PO2: Learners will practice alternative ways to express love and sexuality	2: It will be more special and I will feel good about wanting to have sex until I am in a committed relationship	2: I want to feel like an adult	2: Peer group influences – could go in either direction, but would often encourage early sexual debut		2. I will use other means than intercourse to show my affection (kissing, petting, holding hands etc.)
	PO3: Learners will develop interpersonal communication skills to discuss sexuality	3: I won’t have to worry about getting pregnant or getting an STI/HIV	3: I need the money/gifts that I get from having sex, or my parents need them	3: Parental monitoring and control – would most often discourage early sexual debut		3. I will communicate to my boy/girlfriend that it is a good idea to wait to have sex
	PO4: Learners will avoid potential risk situations in which they might end up having sex or being forced to have sex		4: My boy/girlfriend will dump me if I don’t have sex	4: Gender-specific norms – more important for boys than for girls to be able to brag about early sexual debut		4. I will identify and try to avoid situations which make it difficult to refuse sex
	PO5: Learners will avoid alcohol and drug use		5: I want to prove my love to my boy/girlfriend			5. I will avoid using drugs or alcohol which make it difficult to refuse sex

An overview of topics covered, objectives and sample activities of the school-based component of the interventions is shown in Table 2. The differences in content and structures reflect differences in foci, but also differences that stem from variations in culture and context across sites.

**Table 2:** Topics covered during sessions with students for all four sites

**Topic (number of sessions)**	**Objectives**	**Sample activity**
**Cape Town (Western Cape)**		
Values and aspirations (1)	• Meet the facilitator and learn about the programme	Students complete a worksheet involving the design of their own “roadmap” to direct their lives towards their chosen goals and participate in a group discussion about relationships and their place in the roadmap.
	• Identify personal values and aspirations including how they want to treat people and be treated	
Assertiveness and communication (2)	• Identify four styles of communication and their consequences	Students practice construction of an assertive message to convey their wish to a sexual partner, or a friend.
	• Practice assertive communication skills in the context of sexual decision making	
Gender and power (2)	• Differentiate the concepts of sex and gender.	Class discussion about student’s own experiences at home of gender norms and gender inequality.
	• Critically analyse the dominant social ideas about gender power and roles.	
	• Explore the kind of man or woman they want to be.	
Relationships (6)	• Identify the characteristics of a caring relationship	Students read a locally-developed photo-novella and discuss the relationship problems faced by the characters “John and Janine”: alcohol, lack of communication, pressures to have sex when they are not ready and violence.
	• Identify the qualities of an intimate partner they value	
	• Identify and respond to relationship problems,	
	• Develop skills to end relationships respectfully and safely	
Sexual decision making (4)	• Learn about positive and negative consequences of having sex	Students complete a worksheet analyzing the reasons young people report they regretted their first sex, and they develop a set of personal criteria for assessing their own readiness to have sex.
	• Develop action plans to prevent having sex when they are not ready	
	• Identify the behaviors that put them at risk of HIV, STIs and pregnancy	
	• Critically analyse the risks of multiple partnerships, intergenerational partnerships, and transactional sex	
	• Develop skills to use a condom	
Violence in different contexts (4)	• Recognize types of relationship violence and warning signs	Students read a story depicting a scenario in which a girl is forced by her boyfriend to have sex. They identify the underlying factors, the triggers and the opportunity factors leading to intimate partner violence.
	• Understand the reasons people use violence and control to manipulate others	
	• Reflect on their own values and aspirations in relation to violence	
	• Understand the laws related to violence and sexual violence, and the legal support services	
	• Demonstrate risk monitoring and safety planning skills	
Support (1)	• Develop an understanding and empathy toward victims of violence	Students read a story depicting a scenario in which a girl is forced by her boyfriend to have sex. Students discuss issues of power, blame, responsibility and human rights violations.
	• Understand the importance of seeking help for violent experiences, the ways and places to get help, and how to support friends	
Creating lasting change (1)	• Consolidate and share what they have gained from the programme	Students complete and discuss a worksheet focusing on “What am I going to do to be more respected and respectful?”
	• Reflect on their ability to act as agents of change within their schools and communities	
**Dar es Salaam**		
Self awareness (4)	• Be able to set and demonstrate commitment to life goals	Students formulate a “dream tree” and visualise where he/she is and where he/she wants to be in future and make a life goal map.
	• Understand steps to follow to achieve life goals	They group themselves according to their goal with regard to future profession. Each group discusses how to reach that particular goal.
	• Understand factors that might inhibit the achievements of life goals and find ways to overcome them	
My sexuality (3)	• Differentiate between sex and sexuality	Students divide in small groups and identify similarities and differences in values.
	• Identify sexual values	
	• Explain meanings and levels of sexual expression	
Relationships (2)	• Be able to make healthy decisions about engaging in relationships	Students are divided into small mixed groups of boys and girls. Each group discusses one question about relationships.
What influences my sexuality (1)	• Be able to understand the influences on sexuality	Students brainstorm and list down what influences sexual behaviour.
Risk taking, sexual behaviors and consequences (5)	• Recognise sexual risk behaviours amongst youth	Students divide in groups and identify pictures showing different sexual risk behaviours. Students also list effects of engaging in risky sexual behaviours. Students read a letter and explain 4 challenges faced by the writer.
	• Differentiate sexual risk behaviours and safe sexual behaviours	
Self-protection (1)	• Understand the importance of using condoms consistently and correctly	Demonstration of putting on a condom using a model. Students list places where they can get condoms and correct information about condoms and their use. Students debate whether adolescents should use condoms. Students discuss different scenarios that could be challenging for them in using condoms and come up with suggestions for overcoming them.
	• Recognise different types of condoms (male and female)	
	• Reduce misconceptions about condom use.	
Decision making skills* (4)	• Demonstrate decision making skills	Peer modelling: The peer leaders randomly choose five students to give their experiences on decision making and how such decisions made an impact in their lives.
	• Use the skills in different situations including resisting coercion or temptations to engage in sexual practices	
Puberty* (2)	• Recognise emotional changes which are normal during puberty and learn to respond in healthy ways	Students identify one emotional feeling that they experienced during puberty and write on a strip of paper. Learners link the strips together to appreciate that there are a range of emotions connected with puberty.
Self-protection* (3)	• Demonstrate different skills aimed at self- protection	Students divide in small groups and list illicit drugs used by people and their effects at different levels (individual, family, school and community).
		Students discuss different types and meanings of sexual violence. Students divide in small groups to read different sexual risky scenarios and role-play safety strategies.
**Limpopo (Mankweng)**		
Self-concept formation and self-motivation (3)	• Understand the meaning of and factors influencing self-concept and self-motivation	Ask students to list and categorize things they like and things they dislike. Request students to write a biography about themselves. Each student should share his or her write-up with another student in class. Have them tell the class the biography of this other student.
	• Be aware of their own individuality and know that they are different from others	
	• Understand and develop positive attitudes towards goal setting	Students work individually on setting goals, fill out worksheets and report back to class.
Development of self and society (3)	• Understand physical changes, gender differences and sexual and reproductive system	Students discuss challenging aspects of puberty and learn to appreciate the most exciting aspects. Discussions in same sex groups on what changes they experience.
	• Develop skills to refuse sexual intercourse	
Understanding sexual health (3)	• Understand reproductive health, how a woman gets pregnant and STI transmission	Discuss the different STIs, use of worksheet. Discuss difficult concepts and explain modes of transmission.
	• Know how to avoid HIV infection by using condoms, develop a concept of healthy sexuality	Use of problem-solving skills to determine what choices could be made when a teen couple discovers the girl is pregnant.
Protection against pregnancy and STIs including HIV and AIDS (3)	• Identify types of contraceptives, and learn how to procure and use	Students are split into groups. One contraception method is assigned to each group. Each group is provided with a birth control fact sheet, which they must study, then fill in the appropriate section of a contraceptive Methods Chart and present to the whole class/group.
	• Value the consequences of delaying sexual debut	Introduce the idea of abstinence or delaying sex. Role play a common situation between two young people while thinking about reasons why they should delay their sexual debut.
Social influence (3)	• Understand and dispel myths and misconceptions pertaining to sexuality	Students identify and discuss common myths and misconceptions pertaining to sex, and distinguish truth from fiction.
	• Identify social norms related to sexuality and how these influence sexual behaviour	Role play on taking responsible decisions.
**Kampala**		
Change (1)	• Identify the physical, emotional, social and psychological changes that occur during adolescence	Students grouped by gender - boys alone and girls alone - are asked to identify the changes that girls and boys experience as they grow up. The changes are then categorized into: emotional, physical and social changes.
	• Explore how these changes affect adolescent’s feelings about the opposite sex	Brainstorming on the myths about puberty changes and provision of facts about puberty.
	• Explore myths and anxieties about adolescence	
Coping strategies (2)	• Examine their personal feelings about puberty and the impact on self esteem	Students complete questionnaires about “What do I think about myself”, and examine their self-esteem and identify areas for improvement.
	• Identify strategies for coping with negative feelings about puberty	
	• Develop healthy coping strategies when experiencing different rates of physical, emotional, sexual and social development	Students provided with negative thoughts such as: ‘I have no friends in this class’
		‘I will never be successful’ and asked
		to brainstorm self-talk responses such as:
		‘I spend time with others during break’
		‘With practice I can do it’.
Choices (1)	• Define and review their values and the importance of living by those values	Students are provided with a list of value statements from which they identify their values.
Personal decision making (1)	• Examine the influences on personal decision making	Students are guided into developing a personal timeline. Along the timeline, they mark off accomplishments they would like to achieve and events such as the ages at which they would like to:
	• Examine their personal life goals and decisions	
	• Explore the impact of becoming sexually active on their plans	• start their first job, and the occupation.
		• become involved in a long term relationship or marriage
		become a parent and the number of children.
Relationships (4)	• Clarify understanding of the term relationships and the meaning of dating	Students work in groups and discuss scenarios about the different kinds of relationships. They come up with suggestions for dealing with the situations described in the scenarios and present to the class.
	• Identify the characteristics of healthy and unhealthy relationships	
	• Practice skills for building, maintaining and enhancing healthy relationships	
	• Examine various attitudes , values and behaviours for developing meaningful interpersonal and dating relationships	Students act out a skit on dating relationships. After the skit, they form groups and engage in an imagination exercise. They imagine what they would do in the situations presented. Group imaginations are then presented to the class for further discussions.
	• Identify various responsibilities and consequences associated with involvement in a sexual relationship	
	• Determine those activities and behaviours that are high risk for STIs or pregnancy	
Abstinence (2)	• Examine abstinence and argue in favour of, or against it	Role plays demonstrating how characters can negotiate to remain abstinent or postpone sexual activity followed. by a discussion on:
	• Develop skills that promote abstinence and help to postpone sexual activity	• Pressures
		• Refusal skills
		• Alternative actions
		• Feelings of the students as they watched the role play.
Use of substances (1)	• Consider the influence of substances on sexual decision making	Students discuss a scenario about a student who drank excessively and was sexually assaulted by a class mate at a party.
	• Identify the long term and short term effects of substances on sexual decision making	
Influence of the media (1)	• Understand the influence of the media and culture	Students examining an advertisement and discuss a set of questions about the obvious and subliminal messages conveyed in the advertisement.
	• Identify the effects of social influences on sexuality, gender roles and equity	
STIs (2)	• Define STI/HIV and discuss why it is important to learn about STIs/HIV	In groups students discuss and complete scenarios about STI transmission and consequences of infection, including untreated infections.
	• Develop and practice STI prevention skills.	

*These session in the Dar es Salaam intervention will be peer-led.

### Western Cape intervention

South Africa has high levels of violence against women, and sexual assault and intimate partner violence contribute to increased risks for HIV infection [26]. Adolescent sexual relationships are marked by a high incidence of violence, particularly in first sexual encounters [27], and 15% of high school students report having been assaulted by their boyfriends/girlfriends [28]. In Western Cape, 40% of young adolescents in relationships have experienced intimate partner violence, and they are more likely to have an early sexual debut and to be coerced into sex than those who do not experience such violence [29]. Schools are among the most common locations for sexual assault of students [30]. Our focus on intimate partner violence and HIV was a response to overwhelming evidence in South Africa and elsewhere that interpersonal partner violence is a leading cause of reproductive health problems, including HIV, sexually transmitted infections and unwanted pregnancy [31].

The Western Cape intervention aims to prevent interpersonal partner violence and HIV by implementing an 21-lesson PREPARE curriculum adapted from the Respect4U programme (http://www.mrc.ac.za/gender/respect4u.htm). An overview of topics covered, objectives and sample activities is shown in Table 2. These interactive lessons for young adolescents are designed to change norms that legitimate male control in relationships and gender power inequities, increase the agency of young women in regard to relationships and sexuality, improve communication to prevent the use of violence in relationships, and increase motivation and skills to delay sexual debut and increase condom use.

The curriculum focuses on changing the unequal position of women and men in relationships and in society, and ideologies of male superiority that legitimise control of women by men. These are seen as the underlying factors contributing to the prevalence of interpersonal partner violence and they prevent women from negotiating safer sex practices [32]. For example, the Gender and Power Unit comprises two lessons in which students learn the difference between the concepts of “sex” and “gender”, discuss their own experiences of gender norms and pressures, and learn to identify inequities and the abuse of power in relationships. The Relationships Unit starts with lessons in which students define the qualities of caring relationships, and then fill in a worksheet specifying the characteristics of their ideal partner using the metaphor of a cake (essential partner qualities are the basic ingredients, the “nice-to-have qualities are the decorative touches, and the qualities that lead to unhappiness are the rotten ingredients). This leads to a discussion of the impact of partner age, and economic status on gender equity and HIV risk in relationships. The lessons that follow focus on sexual decision making within relationships, using a photonovella to spur discussion of questions such as “how does one know when one is ready to have sex?”; “how does one deal with pressure to have sex?”; “how many partners are too many?”; and “how does one end a relationship safely?”.

A curriculum which focuses on an individual’s motivation and skills only is insufficient when features of the environment limit the ability to take individual action in response to what is learned. This was evidenced by the failure of the SATZ Cape Town intervention to impact on adolescent risk behavior in Cape Town. Therefore the Western Cape PREPARE project aims to change the school environment in two aspects. Firstly, we aim to increase adolescent access to sexual- and reproductive health services including condoms, contraception, STI management and pregnancy tests through the establishment of a school-based health service (SBHS). Although there are no known randomized controlled trials of the impact of SBHSs, they are considered to be one of the most accessible and acceptable strategies for delivering health services to young people who need them the most [33]. An evaluation objective, in addition to those already mentioned, is to assess whether SBHSs increase adolescent access to sexual and reproductive health services, and whether adolescents most at risk of HIV, STIs and interpersonal partner violence use the service.

We also aim to change the school environment by reducing sexual violence and increasing feelings of safety by creating a school climate of intolerance towards violence. This is to be achieved through a partnership between teachers, students, parents and police officers focusing on the implementation of a school safety audit, raising awareness of relevant laws concerning sexual violence, and of existing services within the community where support is provided. Through a participatory research strategy called Photovoice students are given the opportunity to take photographs of, and think critically about aspects of their school life, and raise awareness of school safety issues among teachers, parents, police officers, and community stakeholders with the aim of influencing policy and prompting concrete change [34]. An evaluation objective in Western Cape (also in addition to those previously mentioned) is to provide evidence about whether such school-level interventions reduce the acceptability and perpetration of sexual and other forms of violence in the school, and increase feelings of safety.

### Dar es Salaam intervention

In order to produce sustained behaviour change, interventions should, in addition to individual level change strategies, also include involvement of groups, social networks and communities [35-37]. Adolescents in particular have been found to be strongly influenced by peer behaviours and norms [38,39].

A recent study in Tanzania indicated parental and peer pressure to be important predictors of early sexual debut among adolescents [40]. Findings from a previous school based intervention suggested that communication with teachers about HIV and sex in Dar es Salaam was associated with delayed self-reported sexual initiation among adolescents after adjusting for potential confounding factors [41]. Process evaluation findings from this earlier intervention showed that more than 16 hours of classroom sessions were not feasible within the school curriculum in Dar es Salaam primary schools. Large class size restricted participatory teaching and learning approaches and teachers were uncomfortable providing information on condom use to young adolescents (Khalifa Mrumbi, Muhimbili University of Health and Allied Sciences, personal communication, July 2013). With these findings in mind, the formative phase of the Dar es Salaam PREPARE comprehensive intervention focused on gaining better understanding of peer-to-peer communication on issues related to sexuality and sexual health, the feasibility of peer-led components for an intervention, and strategies for building working relationships between schools and youth friendly health services.

Formative studies and meetings with teachers informed the objectives, the content and the approach of the Dar es Salaam PREPARE intervention. The health promoting aspects also included learning to recognize symptoms of common sexually transmitted infections (STIs), being able to describe location of and type of services provided by youth friendly health services and being able to demonstrate correct condom use. Interpersonal and contextual factors were also addressed, for instance promoting learners’ ability to communicate on sexuality and discuss sexual health with peers, parents, teachers and health care providers.

The curriculum-based intervention consists of three components, two implemented in school by trained teachers and peer-educators and one implemented by health care providers during class visits to youth friendly health service clinics. An overview of topics covered, objectives and sample activities is shown in Table 2.

The in-school components include nine lessons, three of which are peer-led, taught over 19 hours (11 hours teacher led sessions and 8 hours peer-led sessions). The in-class sessions will be integrated in the primary school science curriculum and taught as 16 interactive teaching and learning sessions suited for large classes with some didactic lessons, each session lasting for 40–80 minutes. They build on a previous intervention, evaluation of which showed effects in delaying sex initiation [12].

Peer-led lessons will be implemented over nine weeks (once a week), each session lasting 60–90 minutes. The sessions are interactive and teachers are available and can offer support when needed. These sessions are part of an after-school life-skills training curriculum. The individual skills sessions focus on experiential learning using scenarios derived from the formative phase narrative’s, role-play, and drama, and are aimed to empower learners to work with adults in order to change the secrecy surrounding sexuality and sexual health communication. Furthermore, it is a goal to facilitate communication of healthy sexuality messages and actions to peers and adults in young persons’ lives.

In order to address external issues such as access to needed information and services to foster healthy sexuality, the third component aims to promote collaboration between schools and youth friendly health services, and to increase the possibilities for access to sexuality- and reproductive health information and services for young adolescents. Such services include access to condoms, contraception, STI management and HIV and pregnancy tests services that are currently not available in the primary school system. This component included feedback from panel discussions that involved parent representatives, health care providers and teachers during program development.

### Limpopo intervention

The Information, Motivation, Behavioural skills model (IMB) [16,42] proposes that HIV-preventive interventions are most effective when they target particular deficits in these three domains. Such deficits (for example, missing or inaccurate information or mistaken beliefs about others’ behaviour patterns) can be identified using elicitation research. Three focus groups (high school students aged 15–17) including only young women, three including only young men and one mixed focus group as well as 7 individual interviews were conducted to explore young people’s views about sexual behaviour, contraception HIV/AIDS and condom use. The focus group and interview data were transcribed verbatim and a thematic analysis undertaken. The results of this analysis were used to develop a questionnaire assessing culturally-relevant aspects of IMB and focusing on issues identified during the thematic analysis. A questionnaire survey was carried out among high school students in order to identify important beliefs which should be targeted in the intervention. Identification of intervention targets was based on analysis of 893 completed questionnaires, together with discussions with local experts (school teachers, University of Limpopo departments, Limpopo Provincial Department of Education) on key cultural beliefs relevant to HIV-prevention.

Twelve key intervention targets corresponding to the three main categories of factors influencing behavior within the IMB framework [16] were identified:

Information

I1. A minority of young people are having sex.

I2. HIV is a primarily a sexually transmitted disease.

I3. Condom use is safe.

I4. Consistent condom prevents sexual transmission of HIV.

I5. Anal sex is unusual and very risky.

Motivation

M1. Having sex is not so important for a young person.

M1. Young people in S Africa can avoid HIV infection.

M3. Others are using condoms – why aren’t you?

M4. It is not OK to force someone you know to have sex (with or without a condom).

Behavioural skills

B1. Carry a condom if you want to have sex.

B2. It is good to ask a sexual partner to use a condom.

B3. Unprotected sex does not mean commitment or love. Say, “No-condom-no-sex”.

Discussions between teachers and public health specialists led to an integration of the 12 messages above into a programme which also focused on identifying and changing culture-specific beliefs which may undermine HIV-preventive information, motivation and action. The intervention targets identified through the IMB elicitation research were integrated into pre-existing health education programmes in schools which also focused on self-esteem and biological aspects of disease transmission.

The programme will be delivered in 5 three-hour school “units” to grade 8 children who are typically aged 12–14. An overview of topics covered, objectives and sample activities is shown in Table 2.

The first topic focuses on personal and social identity including relationship with peers, experiences of violence and traditional and culture-specific religious beliefs. This unit will also explore health motivation and motivation related to achievements and goals in life.

The second topic (Self and Society) explores social identity in relation to what others are doing and thinking, especially in relation to sex. Normative feedback is used to clarify that most young people of this age are not having sex or engaging in HIV-risk activities. The unit involves discussion of maintaining “girlfriend/boyfriend” status and “being in love” without becoming sexually active. This unit also explores gift giving as normative pressure on girls to have sex and how this can be resisted.

The third unit deals with more difficult topics including coercive sex and sex and violence. It asserts girls’ right to wear “sexy” clothes, their right to say, “no”, the need to be assertive and to protect against male violence. A series of culture-specific beliefs which may undermine anti-violence, contraceptive and HIV-preventive motivations are named and challenged.

The fourth topic covered is contraceptives and HIV-preventive behavioural skills.

This unit emphasizes the availability and safety of condom use and advocates getting and carrying condoms when thinking about sex and using condoms during sex. The unit also explores monogamy and the advantages of being faithful to one partner. Specific condom use behavioural skills are discussed and anti-condom culture-specific beliefs named and challenged.

Finally, the fifth topic (social influence) explores social identity construction and social influence processes including religious and peer influence. The importance of friends, community and family are acknowledged and the relationship between these social values and sexual behaviour and HIV prevention is explored.

### Kampala intervention

Parent–child communication about sexuality has been shown to encourage (i) delayed onset of first sexual activity [43,44], (ii) increased sexual abstinence [43,44] (iii) increased partner communication [45] and (iv) practice of safer sex, if sexually active, through condom use [44-46].

Data from a Ugandan adolescent survey showed positive associations between parent- child communication and delayed sexual activity among female adolescents. Positive associations were also identified between parent – child communication with contraceptive use at last sex among adolescents of both genders [47]. Parents might therefore be in a position to protect young adolescents from negative health outcomes associated with sexual risk-taking and related risk behaviors.

Our focus on promoting parent-adolescent communication on sexuality is a response to the growing evidence that Ugandan parents do not communicate with their adolescent children on sexuality because it is regarded as a taboo in many Ugandan cultures. If such communication ever takes place, it is often negative, vague and authoritarian [48,49]. Parents are uncomfortable communicating openly with their children on issues relating to sexuality and often use fear-arousing approaches that emphasize the negative consequences of engaging in sexual activity [48]. This serves as an additional barrier to open and frequent communication between children and their parents on issues relating to sexual and reproductive health [50].

In many traditional Ugandan cultures communication on issues relating to sexuality is delegated to other members of the extended family. Among the Baganda, for example, paternal aunties referred to as ‘sengas’ are traditionally responsible for communicating with their nieces about issues relating to sexuality [51]. This assumes that the sengas live in the same location and are able to communicate frequently, but this is not always the case, especially in urban areas. This tradition has become difficult to maintain due to changes in family structures and dynamics. Studies conducted as part of the PREPARE Kampala formative research showed that adolescents desired to talk to their parents on sexuality issues.

The design of the intervention was based on the formative research which was conducted in 4 secondary schools in Kampala and Wakiso districts. It involved 11 focus group discussions with students and parents, 10 key informant interviews with teachers, school administrators and opinion leaders and a survey of 425 senior two students. The analysis focused on attitudes, content, frequency and comfort with adolescent-parent communication on sexuality and identified attitudes and gaps in knowledge and life skills.

The intervention consisted of three components. The first component is a 14 times 90 minutes lesson classroom based component that integrated sexual- and reproductive health content into English and Christian Religious Education lessons. This component also modified delivery strategies by introducing more learner centred approaches. Teachers will be trained in pedagogical skills in preparation for implementation of the classroom based component. The Christian Religious Education lessons were selected based on the existing standard national curriculum for Senior 1, which covers topics relating to changes during adolescence and other changes experienced by learners in their new environment, new friends and relationships. The lessons are designed to increase motivation and skills to delay sexual debut. The English core curriculum for Senior 1 includes activities designed to build confidence and self-expression skills among students through debates, role plays and writing exercises. An overview of topics covered, objectives and sample activities is shown in Table 2. Condom education was offered as an extracurricular 2-hour activity aimed to increase students’ knowledge and positive attitudes towards condoms.

The second component is homework. For each lesson there is a corresponding homework assignment that students are expected to discuss and complete with their parents/guardians. The third component is workshops for parents. Parents will be mobilized through schools and trained in communication skills and assisted to improve their parenting skills in 3 one-day workshops. The first workshop will precede the implementation of the classroom and homework components. It will focus mainly on giving the parents an overview of the PREPARE project and prepare them for the homework component. The second workshop will be conducted half way through the intervention and focus mainly on communication and parenting skills. The third workshop comes towards the end of the intervention and will focus on sexually transmitted infections including HIV and prevention measures. All workshops are planned to be highly interactive with parents sharing their experiences in the 2nd and 3rd workshops. PREPARE project staff will attend all the workshops to assist the facilitators in responding to the parents’ questions.

## Methods

In Western Cape and Limpopo, students in grade 8 (first year of high school) (age 13–14) are included in the study, in Dar es Salaam students in grades 5 and 6 (age 12–14) are recruited, and in Kampala students in Senior One – the first year of secondary school (age 12–15) – are invited to take part.

All four evaluations of interventions within the PREPARE project are based on cluster randomized designs [52] with schools as the unit of randomization. In Western Cape and Dar es Salaam there will be one pre-test and two post-tests (shortly after the delivery of the interventions and 12 months after baseline). In Kampala and Limpopo there will be one pre-test and one post-test data collections. All power calculations are based on power calculators where the effect of allocating clusters (school classes) instead of individual students is adjusted for, such as the University of Aberdeen Sample Size Power Calculator [53,54]. Intraclass correlations (ICCs) with schools as units of clustering are calculated based on data from previous data collections, and sample size estimates are based on information about ICCs as well as information about cluster size (number of students in relevant grades). Number of schools involved in the studies varies from 22 in Kampala to 40 in Western Cape. Stratification and matching techniques are applied differently in different sites, according to local circumstances.

During the pilot phases of the projects, a wide range of methods were applied, including personal interviews, focus group interviews and reviews of previous studies.

Questionnaires with pre-coded response categories are chosen as the most central approach to collecting data for the evaluation of interventions. A distinction is made between a set of core questions, which are common across sites, and special focus questions, which are unique to each site. Questionnaires are piloted in all sites. The core questions consist of those that are common for the two sites with the most comprehensive interventions (Western Cape and Dar es Salaam). In the Limpopo and Kampala sites, a smaller selection of core questions is included. Special focus questions are piloted only by the site in which they are going to be used. The minimum number of cases in each pilot data collection is set to 200. The language used during the development of questionnaire items is English. For versions in other languages than English (Swahili, Sepedi, Luganda, Afrikaans, and isiXhosa) translations are followed by re-translations and careful examination of discrepancies between original and retranslated versions.

All data collections will take place in schools and are carried out by trained interviewers from the research teams. Teachers will not be present in class during data collection. Each team will follow a standard procedure that is designed to provide all students with necessary background information, constitute a neutral and open setting, and avoid collaboration among students during the data collections. As advocated by Mathews and associates, active consent from parents has not been established as a norm for all sites [55]. Active consent from parents is required in three sites, Cape Town, Limpopo and Kampala. Passive consent is approved and will be practiced in Dar es Salaam. Active consent from students will be requested in all sites. In one site (Dar es Salaam) signed communal consents from head teachers and chairs of school parent committees will be also required.

### Western Cape

The Western Cape sample size was calculated for one of the primary trial outcomes, the annual incidence of sexual debut. We used estimates from the Western Cape SATZ study [11,12] to make assumptions about the prevalence of students reporting at baseline to be virgins (83%), the background incidence of sexual debut during the PREPARE follow-up period of 1 year (17%), the ICC (0.06) and attrition after one year (20%). We used the Hayes and Bennett formula [52] and calculated that to show a 50% relative reduction in incidence of sexual debut (17% in control schools and 8.5% in intervention schools) with 80% power for a 2-sided test with a significance level of 5%, we would need 19 schools in each arm, with 62 “virgins” at baseline in each school. We sampled 20 schools in each arm to allow for one school per arm to drop out of the study. An attrition of 20% is an optimistic estimate, in an environment where 55% of Grade 8 Western Cape students drop out before completing Grade 12. We made this assumption on the basis of our plans to improve on the SATZ attrition (26-28%) by taking the contact details of students and allocating resources to attrition management.

We assumed the Grade 12 pass rate at a school is an indication of the climate and functioning of the school and its potential ability to benefit from the PREPARE programme. Pass rate is correlated with the amount of school fees charged, indicating it is also a reflection of socioeconomic status. Using the database of public high schools in the Western Cape we excluded schools with Grade 12 pass rates below 40% (indicating their inability to deliver even on their core mandate) and above 97% (indicating well-resourced schools already able to offer students the types of interventions proposed by PREPARE). We also excluded schools in two of the 8 districts situated far from Western Cape, and schools with other HIV prevention trials. Then we stratified schools into two strata of equal size based on Grade 12 s pass rate.

To ensure allocation sequence concealment, a statistician at the Medical Research Council, who did not have any knowledge of the schools, allocated the schools within each stratum to intervention and control arms of the study. He ordered the schools randomly within each stratum and then used a random number generator to give each school a number. Within each stratum, he allocated the 10 schools with the lowest random number to the intervention arm, and the 10 with the highest to the control arm. Some of the schools in the sample have large numbers of Grade 8 students, and in such cases, we selected classes randomly to obtain a sample of at least 75 students.

The Western Cape instrument comprises a paper questionnaire in three languages, printed in full colour in an adolescent-friendly format resembling a “teen magazine” rather than an examination paper. It was piloted and tested during 2011.

A process evaluation will monitor the quality of implementation of the intervention and student uptake of, and participation in it. Process data will be collected through observations of intervention sessions, interviews with the intervention facilitators and focus groups with Grade 8 students.

### Dar es Salaam

The Dar es Salaam site power calculation was based on expected effects of the intervention on sexual debut. According to findings from the SATZ project, 4.8% of adolescents aged 12–14 years in Dar es Salaam were sexually active at baseline [12]. Our pilot results indicated a non-response proportion of 7%. We assumed that the baseline rate of sexual debut will be about the same in this study and that the PREPARE intervention would reduce the proportion of adolescents becoming sexually active by 2.0 percentage point (from 5.0% to 3.0%), corresponding to a 40.0% reduction. The ICC was set to 0.01. To achieve 80% power with a significance level of 0.05 in a two-sided test to detect that change, we would need 38 schools (19 schools in each arm) with 153 learners per school, totalling to 5814.

We listed all schools in the selected Kinondoni District (N = 138) and excluded schools without standard 5 and 6 (N = 6), schools that were privately owned (N = 3) and all special schools for disabled children (N = 8). Six schools served as pilot-schools. All the remaining schools (N = 115) were stratified in to two strata based on number of students and location (urban and semi-urban). All the schools were then matched by size and urban pairs and semi-urban pairs were generated. A total of 19 pairs were randomly selected. To ensure equal representation, numbers of urban and semi-urban pairs were selected proportional to the total number of pairs from each location. From each pair, one school was randomly allocated to the intervention group and the other to the control group.

In each intervention school, two streams from each of grades 5 and 6 were randomly selected to receive the intervention and participate in the evaluation. The same procedure was used in control schools in order to select streams that would be involved in the evaluation. In these streams, consenting students with passive parental consent are screened for eligibility for the evaluation including being able to read a standard statement and being in the age range 12–14 years.

The Dar es Salaam site questionnaire comprises a learner administered paper questionnaire in Swahili. Process evaluation will be conducted using observation of at least two of each classroom based, peer led and youth friendly clinic visit sessions; collection of narrative data to explore experiences and challenges during implementation from teachers, head-teachers, parents, health care providers and learners; and determination of percentage of scope covered from a random sample of learners workbooks to identify areas where content reflected in workbooks may have been missed.

To ensure data quality and participation, project staff will supervise the whole data collection process keeping track of the accrual rates over time. Repeated call back will be made to schools where large proportions of participants are missing during the initial visit. To evaluate intervention fidelity and quality a process evaluation procedure will be put in place to monitor the quality of implementation of the intervention, student attendance and understanding of the intervention. On-going process data will be collected during the implementation stage through observations of intervention sessions, exit interviews with students, peer educators, and teachers.

### Limpopo

The Limpopo site calculated the sample size using one of the primary trial outcomes, a sumscore based on 15 items (ordered response categories) measuring behavioural beliefs related to sexual behaviours (condom use, delayed sexual transition). With a medium to weak effect size (0.30), cluster size equal to 100, an ICC set to 0.05, significance level set to 5%, and power equal to 80%, we needed 11 clusters (schools) in each arm. With 10% attrition within classes, the number of clusters in each arm remains unchanged. In order to allow for drop out of one pair of schools, it was decided to include 12 schools in each arm. With no attrition there will be 1200 students in each arm. With 10% attrition within classes, there will be 1080 students in each arm.

Training for teachers in intervention schools will take place over two weekends and will be evaluated in terms of increased confidence in delivering intervention unit contents.

Each 3 hour unit is divided into 3–10 key tasks that will have to be completed by the teacher to ensure delivery fidelity. A delivery checklist assessing whether each key task is not delivered has been developed for each unit. These checklists will be completed by intervention teachers after each session and by researchers who will observe 20% of intervention sessions.

Twenty young men and 20 young women who have participated in the intervention will be interviewed at the end of the intervention and asked to complete the key tasks checklist. Teachers, observes and students assessments of delivery will be compared and students interviews will be subjected to thematic analysis designed to detect informational, belief, motivational and confidence in behaviour skills changes. As in the three other sites, control schools will be offered the PREPARE intervention once data collection is completed.

### Kampala

The Kampala site calculated the sample size using one of the primary trial outcomes, a sumscore based on 13 items (four ordered response categories) measuring frequency of parent–child sexuality-related communication. With a medium to weak effect size (0.35), cluster size equal to 50, an intraclass correlation set to .06, significance level set to 5%, and power equal to .80, we needed 11 clusters (school classes) in each arm. Since the average class is expected to have about 55 students, there is room for almost 10% attrition. With no attrition there will be 605 students in each arm. With an attrition slightly lower than 10%, there will be 550 students in each arm. For each student included in the study, one parent or caregiver (gender not specified) will be invited to participate.

Since the target age group for the PREPARE project is 12–14, we aimed at recruiting school students from secondary school (Senior 1), and the geographical areas chosen were Kampala and Wakiso districts. We obtained information on all government aided secondary schools located in these districts (N = 39) from the Uganda Ministry of Education and Sports. Twenty eight schools fulfilled our inclusion criteria of being public, day and mixed.

Twenty four schools were paired based on geographic location (urban, peri-urban and rural). We pre-tested questionnaires in two schools (one pair) leaving us with 11 pairs of schools. The list of matched pairs was forwarded to the University of Oslo PREPARE team who allocated the schools within each pair to intervention and comparison arms of the study using graphpad.com tool for randomization.

From each school, we obtained complete lists of senior 1-students, and with the help of teachers we excluded students living in the boarding-section of the school or in hostels. The sample for each school was set to be proportionate to the number of students eligible for inclusion in the study. Finally, we used systematic sampling to obtain the required sample of students from each school.

A process evaluation will be conducted for the classroom based component. Teachers will be required to complete a brief questionnaire after each lesson conducted which will be collected by the researchers at the end of each week. Researchers will also observe lessons and complete observation checklists that focus on delivery of content and methodology use. For every homework assignment given, students and their respective parents will complete homework evaluation forms.

Focus Group Discussions (FGD) will be conducted at the end of the intervention with 12 students and 12 parents from each of the 11 intervention schools. In addition, FGD’s will be held with teachers from each of the participating schools. The lesson assessments of both the teachers and the researchers will be compared with the students’ experiences or perceptions during the analysis. For the homework assignments, the students’ and parents’ experiences and thoughts will be used to assess their value.

Process evaluation for parent’s workshops will be conducted using written, oral and direct observation by the research assistants. A standardized form will be completed by the workshop facilitators at the completion of each workshop. Process evaluation will also be conducted for the training of trainer’s workshops using a standardized form to collect information on overall quality of the workshops and facilitators’ satisfaction.

## Discussion

Although there are differences in study objectives, there are several important communalities across the four studies. All studies are based on an evidence-driven and theory-based approach to intervention design and evaluation. All interventions involve school students. In all sites interventions are based on formative research which includes the use of individual and focus groups interviews. The evaluation designs are based on cluster-randomization with an intervention group, comparison group, pre-test and at least one post-test. Data are collected with questionnaires to be administered in schools and the instruments used for evaluating interventions share a core of common questions and scales. The development of interventions has borrowed from the intervention mapping approach [19] and has taken place through a process of mutual exchange of information and experiences among all partner institutions. In all sites there is a focus on mediators of programme effects. This includes social cognition factors and processes.

In the past, in the field of health behaviour research, two traditions emerged [56]. In the United States and Europe, health behaviour research to a large extent focused on individual behaviour change, drawing on conceptual frameworks from social psychology and health psychology. In developing countries, on the other hand, until the mid 1990s, health behaviour research was less conceptually and theoretically oriented. The literature was dominated by empirical studies based on biomedical and epidemiological frameworks. The research was strongly disease-focused, and the purpose of studies often was to identify specific determinants of health practices. It was to a large extent oriented towards family and community contexts of behaviours.

More recently, however, social cognition models, which constitute the mainstream of theoretical frameworks in health behaviour research in Europe and the United States, have been applied in developing countries. This has become an issue of controversy. Catherine Campbell [57] is among the critics of the applications of such models in “highly marginalized communities”. According to her, these models conceptualize individuals as rational information processors. Behaviour is seen as determined by a combination of individual factors such as individual action plans, attitudes, and perceived social norms. She admits that such models are sometimes successful in predicting how people behave in relatively affluent countries or groupings. However, she criticizes the application of such models in developing countries because they tend to focus on personal and proximal determinants, neglecting the wider social context. It has even been argued that psychologists, by introducing such theoretical models, have actually hindered HIV prevention efforts in the developing world [58].

Studies have shown, however, that cognitions such as beliefs, attitudes, subjective and descriptive norms, perceived behavioural control, self-efficacy and intentions do predict behaviour cross-sectional as well as prospectively, and that interventions that are at least partly based on social cognition models succeed in influencing behaviour in sub-Saharan Africa [59-61]. One of the purposes of the PREPARE project is to examine the importance of social cognition factors in predicting behaviour in sub-Saharan cultural contexts. This requires development, piloting and use of a number of new scales and revisions of old scales for the measurement of social cognition factors and culture-specific beliefs which may add to the predictive utility of social cognition models applied in African cultures.

The application of systematic approaches for program development such as program matrixing and intervention mapping has resulted in a clear identification of learning objectives. The identification of differences in determinants resulted in differences in learning objectives across sites. This process also resulted in differences and similarities in behavioural change strategies and practical methods. Behaviour change strategies that will be used in all sites include for instance, persuasion, social modelling, reinforcing positive outcome expectations of the HIV prevention actions, and reinforcing self-efficacy and developing concrete action plans. There are also pronounced differences across sites. For instance, because of the salient role of violence in HIV prevention in South Africa, behaviour change strategies and methods to reduce violence are developed in the Cape Town site. In Dar es Salaam, constructive peer-to peer and parental communication on emotional and cognitive changes related to puberty and links with sexual decision making and behaviour will be emphasized. In Kampala, communication between adolescents and parents/caregivers in issues related to sexuality will be emphasized. Since the existence of myths regarding HIV and other culture-specific beliefs were identified as a crucial factor in Limpopo, this aspect will receive substantial attention in this site.

## Conclusion

There are a number of published evaluation studies of school- and community-based interventions in sub-Saharan Africa. Some have been able to demonstrate behavioural effects of interventions (self-reports) [5,62-67]. Some have identified positive effects in specific subgroups of students only [68,69]. Other studies have only been able to show effects on hypothesized mediators [70-73]. The effects of interventions are, however, generally rather small, and increased research efforts have been called for [74,75]. In order to increase the impact of interventions it is important to move beyond the notion of curriculum-focused and school-based interventions. Rather, there is a need for also mobilising parents, health services and other relevant stakeholders. Interventions aiming at changing cognitions and other person factors need to be combined with interventions aiming at changing contextual factors. This is what we try to achieve through the comprehensive PREPARE interventions which take place in Western Cape and Dar es Salaam. There is also a need to create a process of mutual learning among teams involved in sexual behaviour intervention development. This learning process is facilitated by testing and examining specific programme components in various sites, such as the parent-offspring communication component that is focussed in Kampala and the intervention targeting specific dysfunctional beliefs, which is focussed in Limpopo.

### Ethics approvals

The PREPARE study was approved by the Western Norway Regional Committee for Medical and Health Research Ethics. Separate ethics approvals were provided by relevant committees in each of the African sites: Western Cape, South Africa: Human Research Ethics Committee, Faculty of Health Sciences, University of Cape Town; Mankweng, Limpopo, South Africa: MEDUNSA (Medical University of Southern Africa) Research Ethics Committee (MREC); Dar es Salaam, Tanzania: The Senate Research and Publications Committee of the Muhimbili University of Health and Allied Sciences; Kampala, Uganda: School of Medicine Research and Ethics Committee (SOMREC) and the Uganda National Council for Science and Technology (UNCST).

## Competing interests

The authors declare that they have no competing interests.

## Authors’ contributions

LEAa took the lead in designing the study, writing the original project protocol, applying for grants and became the coordinator of the study. He is also the lead author of the present paper. CM, SK, AK, HO, CA, KIK, and HV all contributed to the development of the study design, became work package leaders for various components of the study, provided materials necessary for writing this paper, commented on all versions of the manuscript and have written parts of the text. AW has contributed to the development of the study design, been involved in coordination of the study, worked on data quality control, provided materials necessary for writing this paper, commented on all versions of the manuscript, and written parts of the text. SME have been involved in designing instruments for data collections, been involved in coordination of the study, worked on data quality control, provided materials necessary for writing this paper, has commented on all versions of the manuscript and written parts of the text. All authors have read and approved the final manuscript.

## Pre-publication history

The pre-publication history for this paper can be accessed here:

http://www.biomedcentral.com/1471-2458/14/54/prepub

## References

[B1] UNAIDS UNAIDS report on the global AIDS epidemic 2010 2010 Geneva: Joint United Nations Programme on HIV/AIDS (UNAIDS)

[B2] UNESCO UNESCO strategy for HIV and AIDS 2011 Paris: United Nations Educational, Scientific and Cultural Organization (UNESCO), Education Sector, Division of Education for Peace and Sustainable Development Section of Education and HIV & AIDS

[B3] Green LW Kreuter M Health Program Planning: An Educational and Ecological Approach 2005 4 New York: McGraw-Hill

[B4] de Vries H Dijk F Wetzels J Mudde A Kremers S Ariza C Vitória PD Fielder A Holm K Janssen K The European Smoking prevention Framework Approach (ESFA): effects after 24 and 30 months Health Educ Res 2006 21 1 116 132 16087692 10.1093/her/cyh048

[B5] Ross DA Dick B Ferguson J Preventing HIV/AIDS in young people: A systematic review of the evidence from developing countries 2006 Geneva: World Health Organization 16921915

[B6] Maticka-Tyndale E Wildish J Gichuru M Quasi-experimental evaluation of a national primary school HIV intervention in Kenya Eval Program Plann 2007 30 172 186 17689323 10.1016/j.evalprogplan.2007.01.006

[B7] Kirby D Obasi A Laris BA Ross DA, Dick B, Ferguson J The effectiveness of sex education and HIV education interventions in schools in developing countries Preventing HIV/AIDS in young people: A systematic review of the evidence from developing countries 2006 Geneva: World Health Organization (WHO) 103 150 16921919

[B8] UNAIDS Worldwide HIV & AIDS Commentary, 2010 2013 Geneva: Joint United Nations Programme on HIV/AIDS (UNAIDS)

[B9] Eggers SM Aarø LE Bos AER Mathews C de Vries H Predicting condom use in South Africa: a test of two integrative models AIDS Behav 2013 18 135 145 23392911 10.1007/s10461-013-0423-2

[B10] Eaton L Flisher AJ Aarø LE Unsafe sexual behaviour in South African youth Soc Sci Med 2003 56 1 149 165 12435558 10.1016/s0277-9536(02)00017-5

[B11] Aarø LE Flisher AJ Kaaya S Onya H Fuglesang M Klepp K-I Schaalma H Promoting sexual- and reproductive health in early adolescence in South Africa and Tanzania: Development of a theory- and evidence-based intervention programme Scand J Public Health 2006 34 2 150 158 16581707 10.1080/14034940510032356

[B12] Mathews C Aarø LE Grimsrud A Flisher AJ Kaaya S Onya H Schaalma H Wubs A Mukoma W Klepp K-I Effects of the SATZ teacher-led school HIV prevention programmes on adolescent sexual behaviour: cluster randomised controlled trials in three sub-Saharan African sites International Health 2012 4 111 122 24029149 10.1016/j.inhe.2012.02.001

[B13] Gevers A Flisher AJ Ward C, Dawes A, van der Merwe A School-based youth violence prevention interventions Youth Violence: Sources and Solutions in South Africa 2012 Cape Town: HSRC Press 175 211

[B14] Bandura A Social foundations of thought and action: A social cognitive theory 1986 Englewood Cliffs, New Jersey: Prentice-Hall

[B15] Fishbein M Ajzen M Predicting and changing behaviour 2010 The reasoned action approach. New York: Psychology Press, Taylor & Francis

[B16] Fisher WA Fisher JD Harman J Suls J, Wallston KA The information-motivation-behavioral skills model: a general social psychological approach to understanding and promoting health behavior Social psychological foundations of health and illness 2003 Malden, Massachusetts, USA: Blackwell 82 106

[B17] Elfeddali I Bolmann C Candel MJJM Wiers RW De Vries H The role of self-efficacy, recovery self-efficacy, and preparatory planning in predicting short-term smoking relapse Br J Health Psychol 2012 17 185 201 22107073 10.1111/j.2044-8287.2011.02032.x

[B18] Aarø LE Flisher AJ Wold B, Samdal O Health behaviour in context An ecological perspective on health promotion: Systems, settings, and social processes 2012 Sharjah, UAE: Bentham Science Publishers 24 51

[B19] Bartholomew LK Parcel GS Kok G Gottlieb NH Fernández ME Planning health promotion programmes. An intervention mapping approach 2011 San Francisco: Wiley

[B20] Dillard JP Pfau M The persuasion handbook 2002 Thousand Oaks, California: Sage

[B21] Eagly AH Chaiken S The psychology of attitudes 1991 Fort Worth, Texas: Harcourt Brace Jovanovich College Publishers

[B22] MacKinnon DP Introduction to statistical mediation analysis 2008 New York: Lawrence Erlbaum Assiociates

[B23] Lewis MA DeVellis BM Sleath B Glanz K, Rimer BK, Lewis FM Social influence and interpersonal communication in health behaviour Health behavior and health education - Theory, research and practice 2002 3 San Francisco: Jossey-Bass 240 264

[B24] De Vries H Kok GJ From determinants of smoking behavior to the implications for a prevention programme Health Educ Res 1986 1 85 94

[B25] Abraham C Michie S **A Taxonomy of behavior change techniques used in interventions** 2008 27 3 379 387 10.1037/0278-6133.27.3.379 18624603

[B26] Jewkes R Dunkle K Nduna M Shai N Intimate partner violence, relationship gender power inequity, and incidence of HIV infection in young women in South Africa: a cohort study The Lancet 2010 367 41 48 10.1016/S0140-6736(10)60548-X 20557928

[B27] Flisher AJ Myer L Marais A Prevalence and correlates of partner violence among South African adolescents J Child Psychol Psychiatr 2007 48 619 627 10.1111/j.1469-7610.2007.01711.x 17537078

[B28] Reddy SP James S Sewpaul R Koopman F Funani NI Sifunda S Josie J Masuka P Kambaran NS Omardien RG The South African Youth Risk Behaviour Survey 2008 2010 Cape Town: The Health Promotion Research and Development Unit of the Medical Research Council of South Africa

[B29] Mathews C Aarø LE Flisher AJ Mukoma W Wubs A Schaalma H Predictors of early first sexual intercourse among adolescents in Cape Town, South Africa Health Educ Res 2009 24 1 1 10 18203683 10.1093/her/cym079

[B30] Burton P Merchants, skollies and stones: experiences of school violence in South Africa Monograph Series 2008 Cape Town: Centre for Justice and Crime Prevention

[B31] Jewkes R HIV/AIDS. Gender inequities must be addressed in HIV prevention Science 2010 329 5988 145 147 20616253 10.1126/science.1193794

[B32] Jewkes R Intimate partner violence: causes and prevention Lancet 2002 359 9315 1423 1429 11978358 10.1016/S0140-6736(02)08357-5

[B33] Mason-Jones AJ Crisp C Momberg M Koech J De Koker P Mathews C A systematic review of the role of school-based health care in adolescent sexual, reproductive and mental health Syst Rev 2012 1 49 doi:10.1186/2046-4053-1-49. 10.1186/2046-4053-1-49 PMC3621403 23098138

[B34] Zuch M Mathews C De Koker P Mtshizana Y Mason-Jones A Evaluation of a Photovoice pilot project for school safety in South Africa 2013 Youth and Environments: Child 23(1)

[B35] Kelly JA Community-level interventions are needed to prevent new HIV infections Am J Public Health 1999 89 3 299 301 10076476 10.2105/ajph.89.3.299 PMC1508618

[B36] DiClemente RJ Wingood GM Human immunodeficiency virus prevention for adolescents: windows of opportunity for optimizing effectiveness Arch Pediatr Adolesc Med 2003 157 4 319 320 12695223 10.1001/archpedi.157.4.319

[B37] Jemmott JB Jemmott LS HIV risk reduction behavioral interventions with heterosexual adolescents AIDS 2000 14 Suppl. 2 S40 S52 11061641

[B38] Andrews JA Tildesley E Hops H Li F The influence of peers on young adult substance use Health Psychol 2002 21 4 349 357 12090677 10.1037//0278-6133.21.4.349

[B39] Li F Barrera M Hops H Fisher KJ The longitudinal influence of peers on the development of alcohol use in late adolescence: a growth mixture analysis J Behav Med 2002 25 3 293 315 12055779 10.1023/a:1015336929122

[B40] Mmbaga EJ Leonard F Leyna GH Incidence and predictors of adolescent’s early sexual debut after three decades of HIV interventions in Tanzania: a time to debut analysis PLos One 2012 7 7 e41700 doi:10.1371/journal.pone.0041700 22848571 10.1371/journal.pone.0041700 PMC3407234

[B41] Kawai K Kaaya SF Kajula L Mbwambo J Kilonzo GP Fawzi WW Parents’ and teachers’ communication about HIV and sex in relation to the timing of sexual initiation among young adolescents in Tanzania Scand J Public Health 2008 36 8 879 888 19004907 10.1177/1403494808094243

[B42] Fisher JD Fisher WA Changing AIDS-risk behavior Psychol Bull 1992 111 455 471 1594721 10.1037/0033-2909.111.3.455

[B43] World Health Organization (WHO) Broadening the horizon: balancing protection and risk for adolescents 2002 Geneva: World Health Organization (WHO)

[B44] Dutra R Miller KS Forehand R The process and content of sexual communication with adolescents in two-parent families: associations with sexual risk-taking behavior Aids Behaviors 1999 3 59 66

[B45] Whitaker DJ Miller KS May DC Levin ML Teenage partners’ communication about sexual risk and condom use: importance of parent-teenager communication Fam Plann Perspect 1999 31 3 117 121 10379427

[B46] Neema S Humera Ahmed F Kibombo R Bankole A Adolescent sexual and reproductive health in Uganda: results from 2004 National survey of adolescents Occasional Report No. 25 2006 New York: Allan Guttmacher Institute

[B47] Uganda Bureau of Statistics (UBOS) Statistical Abstract June 2012 2012 Kampala, Uganda: Uganda Bureau of Statistics

[B48] Luwaga LCN Parent-adolescent communication on sexuality in the context of HIV/AIDS in Uganda: An exploratory study 2004 Bergen: University of Bergen

[B49] Katahoire A Muhwezi WW Banura C Kwesiga D Bastien S Klepp K-I Formative research report on parent–child communication on sexual and reprodcutive health in Kamapala and Wakiso districts 2010 Kampala, Uganda: Makerere University, College of Health Sciences, Child Health and Development Centre

[B50] Lofgren J Byamugisha J Tillgren P Rubenson B The perspectives of in-youths in Kampala, Uganda, on the of parents in HIV prevention Afr J Aids Res 2009 8 2 193 200 10.2989/AJAR.2009.8.2.7.859 25875570

[B51] Kibombo R Neema S Moore AM Humera AF Adults’ perceptions of adolescents’ sexual and reproductive health: qualitative evidence from Uganda 2008 Allan Guttmacher Institute: Occasional Report. New York

[B52] Hayes RJ Moulton LH Cluster randomized trials 2009 Boca Raton, Florida: Chapman & Hall

[B53] Donner A Birkett N Buck C Randomization by cluster, sample size requirements and analysis Am J Epidemiol 1981 114 906 915 7315838 10.1093/oxfordjournals.aje.a113261

[B54] Health Services Research Unit, University of Aberdeen Cluster sample size calculator. User manual 1999 Aberdeen, Scotland: University of Aberdeen

[B55] Mathews C Guttmacher SJ Flisher AJ Mtshizana Y Hani A Zwarenstein M Written parental consent in school-based HIV/AIDS prevention research Am J Public Health 2005 95 7 1266 1269 15983279 10.2105/AJPH.2004.037788 PMC1449350

[B56] Coreil J DS. G Health behavior in developing countries Handbook of health behavior research I: personal and social determinants 1997 New York/London: Plenum Press 179 197

[B57] Campbell C ‘Letting them die’ - Why HIV/AIDS prevention programmes fail 2003 Oxford: The International Afria Institute

[B58] Waldo C Coates T Multiple levels of analysis and intervention in HIV prevention science: exemplars and directions for new research AIDS 2000 14 Suppl 2 S18 S26 11061638

[B59] Schaalma H Aarø LE Flisher AJ Mathews C Kaaya S Onya H Ragnarsson A Klepp K-I Correlates of intentions to use condoms among Sub-Saharan African youth: the applicability of the theory of planned behaviour Scand J Public Health 2009 37 Suppl. 1 87 91 19493985 10.1177/1403494808090632

[B60] Protogerou C Flisher AJ Aarø LE Mathews C The Theory of Planned Behaviour as a framework for predicting sexual risk behaviour in Sub-Saharan African youth: a critical review J Child Adolesc Ment Health 2010 24 1 15 35 10.2989/17280583.2011.621067 25865835

[B61] Protogerou C Flisher AJ Wild L Aarø LE Using structural equation modeling to investigate condom use predictors in South African young adults Psychol Health 2012 27 309 310

[B62] Fawole IO Asuzu MC Oduntan SO Brieger WR A school-based AIDS education programme for secondary school students in Nigeria: a review of effectiveness Health Educ Res 1999 14 5 675 683 10510075 10.1093/her/14.5.675

[B63] Shuey DA Babishangire BB Omiat S Bagarukayo H Increased sexual abstinence among in-school adolescents as a result of school health education in Soroti district, Uganda Health Educ Res 1999 14 3 411 419 10539231 10.1093/her/14.3.411

[B64] Brieger WR Delano GE Lane CG Oladepo O Oyediran KA West African Youth Initiative: outcome of a reproductive health education program J Adolesc Health 2001 29 6 436 446 11728893 10.1016/s1054-139x(01)00264-6

[B65] Agha S van Rossem R Impact of a school-based peer sexual health intervention on normative beliefs, risk perceptions, and sexual behavior of Zambian adolescents J Adolesc Health 34 5 441 452 15093801 10.1016/j.jadohealth.2003.07.016

[B66] Erulkar AS Ettyang LIA Onoka C Nyagah FK Muyonga A Behavior change evaluation of a culturally consistent reproductive health program for young Kenyans Int Fam Plan Perspect 2004 30 2 58 67 15210404 10.1363/3005804

[B67] Magnani R MacIntyre K Karim AM Brown L Hutchinson P The impact of life skills education on adolescent sexual risk behaviors in KwaZulu-Natal, South Africa J Adolesc Health 2005 36 289 304 15780784 10.1016/j.jadohealth.2004.02.025

[B68] Stanton BF Li X Kahihuata J Fitzgerald AM Neumbo S Kanduuombe G Ricardo IB Galbraith JS Terreri N Guevara I Increased protected sex and abstinence among Namibian youth following a HIV risk-reduction intervention: a randomized, longitudinal study AIDS 1998 12 2473 2480 9875586 10.1097/00002030-199818000-00017

[B69] Harvey B Stuart J Swan T Evaluation of a drama-in- education programme to increase AIDS awareness in South African Schools: a randomized community intervention trial Fam Plann Perspect 2000 30 2 58 67 10.1177/095646240001100207 10678478

[B70] Klepp K-I Ndeki SS Leshabari MT Hannan PJ Lyimo BA AIDS education in Tanzania: promoting risk reduction among primary school children Am J Public Health 1997 87 12 1931 1936 9431279 10.2105/ajph.87.12.1931 PMC1381232

[B71] Mbizvo MT Kasule J Gupta S Rusakaniko S Kinoti SN Mpaju-Shumbushu W Sebina-Zziwa AJ Mwateba R Padayachy J Effects of a randomized health education intervention on aspects of reproductive health knowledge and reported behaviour among adolescents in Zimbabwe Soc Sci Med 1997 44 5 573 577 9032825 10.1016/s0277-9536(96)00204-3

[B72] Reddy P James S McCauley A Programming for HIV prevention in South African schools. Horizons Research Summary 2003 Washington D.C.: Population Council

[B73] James S Reddy P Ruiter RAC McCauley A van den Borne B The impact of an HIV and AIDS life skills program on scondary school students in Kwazulu-Natal, South Africa AIDS Educ Prev 2006 18 4 281 294 16961446 10.1521/aeap.2006.18.4.281

[B74] Ross DA Changalucha J Obasi AIN Todd J Plummer ML Cleophas-Mazige B Anemona A Everett D Weiss H Mabey DC Biological and behavioural impact of an adolescent sexual health intervention in Tanzania: a community-randomized trial AIDS 2007 21 1943 1955 17721102 10.1097/QAD.0b013e3282ed3cf5

[B75] Paul-Ebhohimhen VA Poobalan A van Teijlingen ER A systematic review of school-based sexual health interventions to prevent STI/HIV in sub-Saharan Africa BMC Public Health 2008 8 4 1 13 18179703 10.1186/1471-2458-8-4 PMC2248569

